# Blockchain based transparent and reliable framework for wheat crop supply chain

**DOI:** 10.1371/journal.pone.0295036

**Published:** 2024-01-11

**Authors:** Muhammad Shoaib Farooq, Zain Khalid Ansari, Atif Alvi, Furqan Rustam, Isabel De La Torre Díez, Juan Luis Vidal Mazón, Carmen Lili Rodríguez, Imran Ashraf

**Affiliations:** 1 Department of Computer Science, University of Management and Technology, Lahore, Pakistan; 2 School of Computer Science, University College Dublin, Dublin, Ireland; 3 Department of Signal Theory and Communications and Telematic Engineering, University of Valladolid, Valladolid, Spain; 4 Universidad Europea del Atlántico, Santander, Spain; 5 Universidad Internacional Iberoamericana, Campeche, México; 6 Universidad Internacional Iberoamericana, Arecibo, Puerto Rico, United States of America; 7 Universidade Internacional do Cuanza, Cuito, Bié, Angola; 8 Information and Communication Engineering, Yeungnam University, Gyeongsan, Korea; Jazan University Faculty of Computer Science, SAUDI ARABIA

## Abstract

The wheat crop that fulfills 35% of human food demand is facing several problems due to a lack of transparency, security, reliability, and traceability in the existing agriculture supply chain. Many systems have been developed for the agriculture supply chain to overcome such issues, however, monopolistic centralized control is the biggest hurdle to realizing the use of such systems. It has eventually gained consumers’ trust in branded products and rejected other products due to the lack of traceable supply chain information. This study proposes a blockchain-based framework for supply chain traceability which provides trustable, transparent, secure, and reliable services for the wheat crop. A crypto token called wheat coin (WC) has been introduced to keep track of transactions among the stakeholders of the wheat supply chain. Moreover, an initial coin offering (ICO) of WC, crypto wallets, and an economic model are proposed. Furthermore, a smart contract-based transaction system has been devised for the transparency of wheat crop transactions and conversion of WC to fiat and vice versa. We have developed the interplanetary file system (IPFS) to improve data availability, security, and transparency which stores encrypted private data of farmers, businesses, and merchants. Lastly, the results of the experiments show that the proposed framework shows better performance as compared to previous crop supply chain solutions in terms of latency to add-blocks, per-minute transactions, average gas charge for the transaction, and transaction verification time. Performance analysis with Bitcoin and Ethereum shows the superior performance of the proposed system.

## Introduction

Wheat crop fulfills the food needs of approximately 35% of the population over the globe with itself and its by-products such as bread, flour, flakes, and other baked products. Wheat holds significant importance for human survival and both its production and supply chain management should be handled efficiently. Especially in the wake of the recent war between Russia and Ukraine, the importance of supply chain management of wheat crops has been elevated. As it holds significant importance for humans, an appropriate system to manage its supply chain is of high interest. The World Health Organization (WHO) has reported many issues related to food safety like traceability, security, reachability, etc. [1]. The farmers have been putting a lot of effort into the production of wheat crops and their motivation is harmed by low revenues during crop harvesting season. In addition, the Government suffers economic losses by spending additional money to import wheat from other countries in the off-season. During the past few years, a variety of initiatives have been taken towards finding the best solution for the traceability of the agriculture supply chain [2, 3]. Existing systems for the agriculture supply chain are predominantly unstructured, especially in underdeveloped countries. Moreover, such systems are non-reliable and lack transparency features. Problems related to food safety have arisen in the past few years such as mad cow disease [4], rice laced with mercury, milk with toxic powder [5], and the scandal of horse meat [6], etc. Also, WHO reports that food infected with hazardous bacteria, viruses, or chemicals can cause over two hundred diseases spanning from diarrhea to cancer [1]. Similarly, war-inflicted areas also add several hazards to wheat crops.

**Fig 1:**
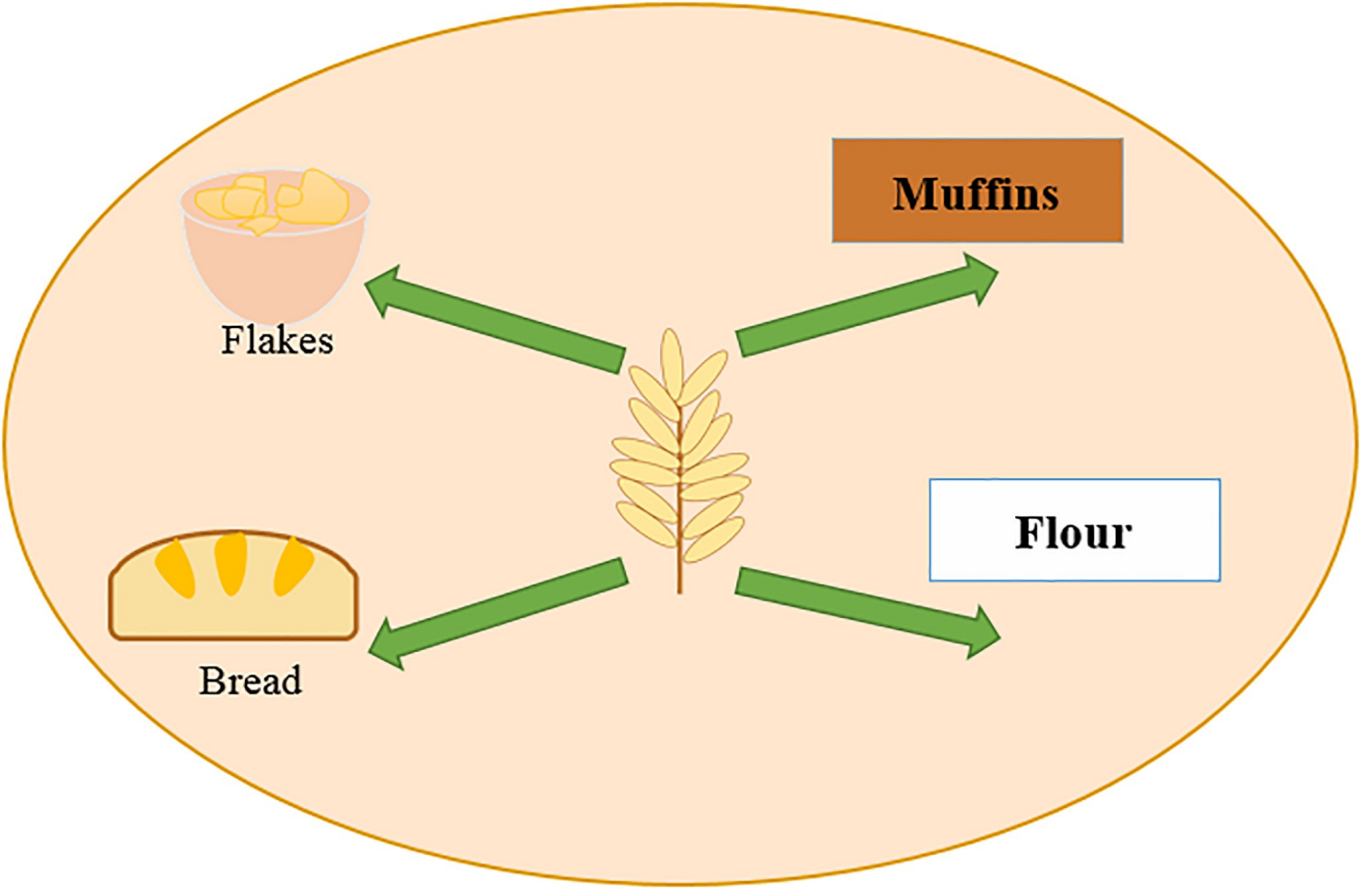
Different by-products of wheat crops.

The wheat crop is the world’s second-largest cultivated crop after the maize [7]. In South Asia, wheat is one of the most important production of crops that cover 12.3 million hectares area of India, 2.2 million hectares area of Pakistan, 0.8 million hectare area of Bangladesh, and 0.5 million hectare area of Nepal [8]. This crop fulfills 35% of human food demand and it increases demand to keep track of the wheat crop supply chain [8]. The wheat crop has been processed to produce many by-products such as bread, flour, flakes, and other baked products as shown in Fig 1 [9].

The wheat crop is in high demand and many farmers prefer to cultivate wheat to earn a good profit, but unfortunately, farmers do not get the real value of the harvested crop. Mostly, investors store wheat at a low price during the season and sell it in the off-season at a higher price, thereby earning substantially compared to farmers. Consequently, the Governments have to give subsidies to its masses which suffers economic losses for spending extra amounts to import wheat crop. Situations like these, also discourage farmers from cultivating the wheat crop [10]. This artificial shortage forces consumers to purchase wheat products at high rates. Such problems have also created trust issues and clashes between supply chain stakeholders due to the poor traceability and reliability of traditional wheat supply chain systems [11].

**Fig 2:**
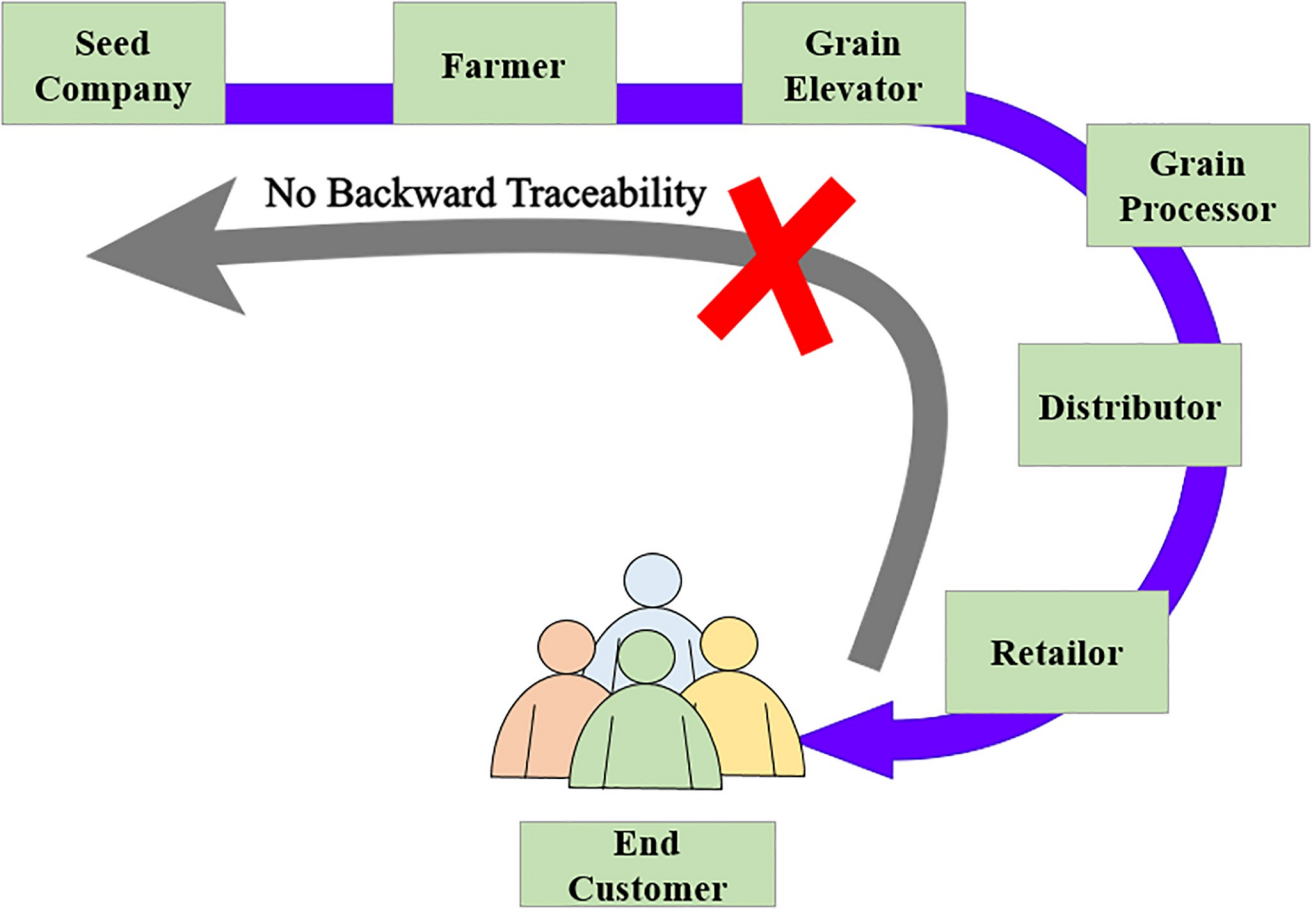
Flow of traditional wheat supply chain.

The major cause of these issues is the lack of transparency in the supply chain of the wheat crop, in addition to traceability and reliability [12]. The farmers put high efforts in cultivating the wheat crop and their efforts have been dispirited with low return-on-investment. The consumers are dissatisfied due to mismanagement of the supply chain systems and buy the expensive wheat crop. A large number of efforts have been made to transform traditional supply chain systems into more robust and transparent supply chain management systems [12]. However, these systems are unable to provide traceability, transparency, reliability, and trust factors between stakeholders. Traditional systems, as shown in Fig 2, have no backward traceability.

A solution to these issues is required to improve transparency and reliability for the wheat supply chain and regulate the price of wheat crops. This problem can be solved by using a blockchain-based transparent and traceable management system for the wheat crop that removes the monopoly of middlemen. For this purpose, it makes use of smart contracts and makes the system decentralized [13]. The decentralization and immutable data are the major features of blockchain that make crop data temperproof and have capabilities to store all transactional and digital data of all supply chain stakeholders related to wheat crops [14]. One of the fundamental components of the blockchain ecosystem is a smart contract that helps in the implementation of business rules approved by blockchain members and consensus protocols [15]. The consensus protocols are the core rules that allow blockchain in decision-making, verifying transactions, and adding new blocks. These consensus protocols and smart contracts increase security, trust level between stakeholders, and cost-effectiveness [16].

**Fig 3:**
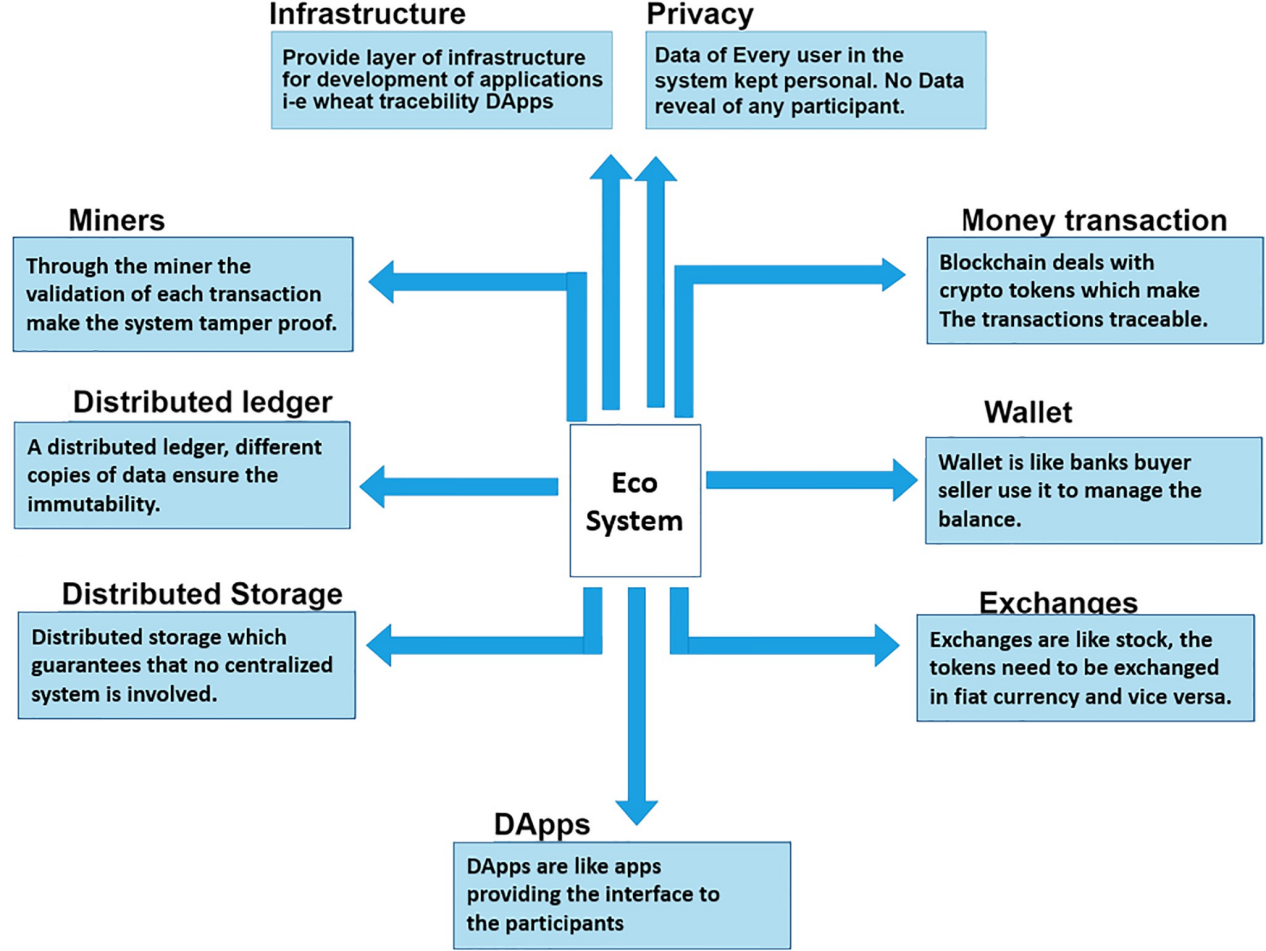
Eco system of the blockchain technology.

The distributed nature of blockchain prevents external parties from illegally accessing, deleting, or altering data, and ensures that the wheat crop supply chain system is transparent and reliable [13, 17]. The trusted entities are allowed to access or change transaction data stored in the blockchain. The data has been added after mining activity that needs a lot of processing power to solve mathematical cryptographical puzzles done by nodes known as miners [12]. Due to distributed structure and security features, blockchain seems to be a perfect technology for creating a wheat crop traceability system. The ecosystem of blockchain with its features is illustrated in Fig 3. The traceability system of the wheat crop depends on different components such as identities of participants, wallets, privacy, and exchanges. The ecosystem of blockchain has exchanges and wallets which enables the flow of cryptocurrency and administration of stores data of transaction with timestamps in a distributed ledger, as shown in Fig 3. An additional component of blockchain is the infrastructure that helps to build DApps that manage communication between stakeholders.

This study adopts blockchain technology to develop a supply chain system for the wheat crop. In this regard, this study makes the following key contributions

- A decentralized blockchain-based system for wheat crop traceability is designed to ensure reliability, transparency, and safety. In addition, the limitation of traceability in the existing system of wheat crops has been resolved.
- A new coin known as wheat coin (WC) has been proposed with the use of Ethereum smart contract in order to control wheat prices. This token has been explored in terms of token generation, total supply, as well as rules for WC, buy, sell, and user authentication. It helps to make the supply chain of wheat crops more transparent for government authorities.
- An initial coin offering (ICO) of WC, crypto wallets, and an economic model is proposed. Also, a smart contract-based transaction system has been devised for the conversion of WC to fiat and vice versa. Data availability, security, and transparency are ensured by developing the interplanetary file system (IPFS).
- Experiments have been performed that show the comparison of the proposed system in terms of latency to add blocks, per-minute transactions, average gas fee, and transaction verification time with other well-known blockchains like Bitcoin and Ethereum.

The remaining article has been structured in the following manner; Section ‘Related Work’ presents the related work about blockchain technologies of different authors in the food supply chain. The proposed framework has been discussed in Section ‘Materials and Methods’. Also, the implementation of the designed framework is elaborated. The experiment and evaluation of the system have been presented in Section ‘Experiments and Results’. Finally, the conclusion of the research is presented in Section ‘Conclusion’.

## Related work

In this section, we have reviewed and highlighted efforts that have been made by different researchers for the agriculture supply chain traceability and reliability. Zhang et al. [18] discussed the issues that have arisen in the safety of food. For this instance, the authors propose a new architecture that is based on the blockchain for the supply chain. The authors designed the storage mechanism as a multimode that combines a chain of storage. The prototype of the system is verified and tested on different scenarios and actual cases. However, the proposed system still has deficiencies regarding traceability and trust in the supply chain.

Caro et al. [19] proposed that the use of Internet of Things (IoT) devices and technologies in the field of agriculture and food has increased which helps to increase the safety of food. The authors defined the assessment of AgriBlockIoT with the help of use cases. Implementation and comparisons are made to evaluate the system in terms of central processing unit (CPU) performance and latency. However, the results are provided only in terms of CPU and latency while many factors are missing. For example, there is no discussion regarding traceability and reliability and the sole focus of the study has been the response time.

The authors investigate issues related to food safety in [12]. Owing to the importance of record-keeping of food and safety, the study proposes a system of traceability for the supply chain of soybeans using the Ethereum blockchain. For security, smart contracts have been used for transactions. However, the study lacks appropriate experimentation to support the supremacy of the proposed approach. Moreover, the Ethereum gas fee is very high which makes the system expensive. Another similar system is presented in [20] that utilizes smart contracts. However, problems like immutability, data privacy, scalability, interoperability, and efficiency are not covered. The use of artificial intelligence-based approaches to resolve the issues of supply chain management is elaborated in [21]. Similarly, a review of supply chain management systems with the help of blockchain-based technologies can be found in [22].

Ghorbel et al. [23] discussed that agriculture supply chain is working in traditional manners throughout the world. Now it is essential to convert traditional systems to digital systems in the agriculture fields. So, the authors created a blockchain-based supply chain system for the olive fields. The developed system is created to monitor the production of the olive fields. The authors used the Ethereum blockchain for the decentralization of the system. For the transactions, the Ethereum Coin has been used which is basically a digital currency. Through this users or authorities easily monitor the transactions.

Yakubu et al. [24] mentioned that safety issues arise day by day to resolve this a traceability system is required. For this author developed a blockchain-based system for rice traceability by using the smart contract which is cost effective. However, they did not include the digital payments and proof of delivery.

Table 1 provides a comparative summary of the discussed research works in the context of security, traceability, smart contract, and type of the system. The above-cited works demonstrate the importance of supply chain management systems to provide transparent, secure, and reliable platforms for the agriculture sector. However, predominantly, the proposed approaches do not provide such characteristics or offer partial solutions to resolve such issues. Specifically, no suitable approach has been presented to address transparency and reliability. Besides that, there is a need for a second layer of protection for government officials to verify and trace back in case of suspected transactions. This study focuses on fulfilling the above-mentioned gaps and providing functionality that is missing in the previous models. The novelty of our work, in addition to providing a safe supply chain solution for the wheat crop, is the addition of a public-private blockchain hybrid solution for officials to monitor supply chain activities or transactions verified by smart contracts without a centralized authority. The proposed system also grants privileges to the government for tracking the wheat supply chain transactions. The proposed system has been designed to preserve all internal and external transaction records within the supply chain. The government can track the transaction for audit purposes and find illegal patterns.

**Table 1:** Comparative analysis of discussed research works.

Reference.	Security	Traceability	System type	Smart contract
[18]	Yes	No	Decentralized	No
[19]	Not mentioned	No	Centralized	No
[12]	Not Mentioned	Yes	Decentralized	Yes
[21]	No	No	Centralized	No
[23]	Not mentioned	Yes	Decentralized	Yes
[24]	Not mentioned	Yes	Decentralized	Yes
This Paper	Yes	Yes	Decentralized	Yes

## Materials and methods

**Fig 4:**
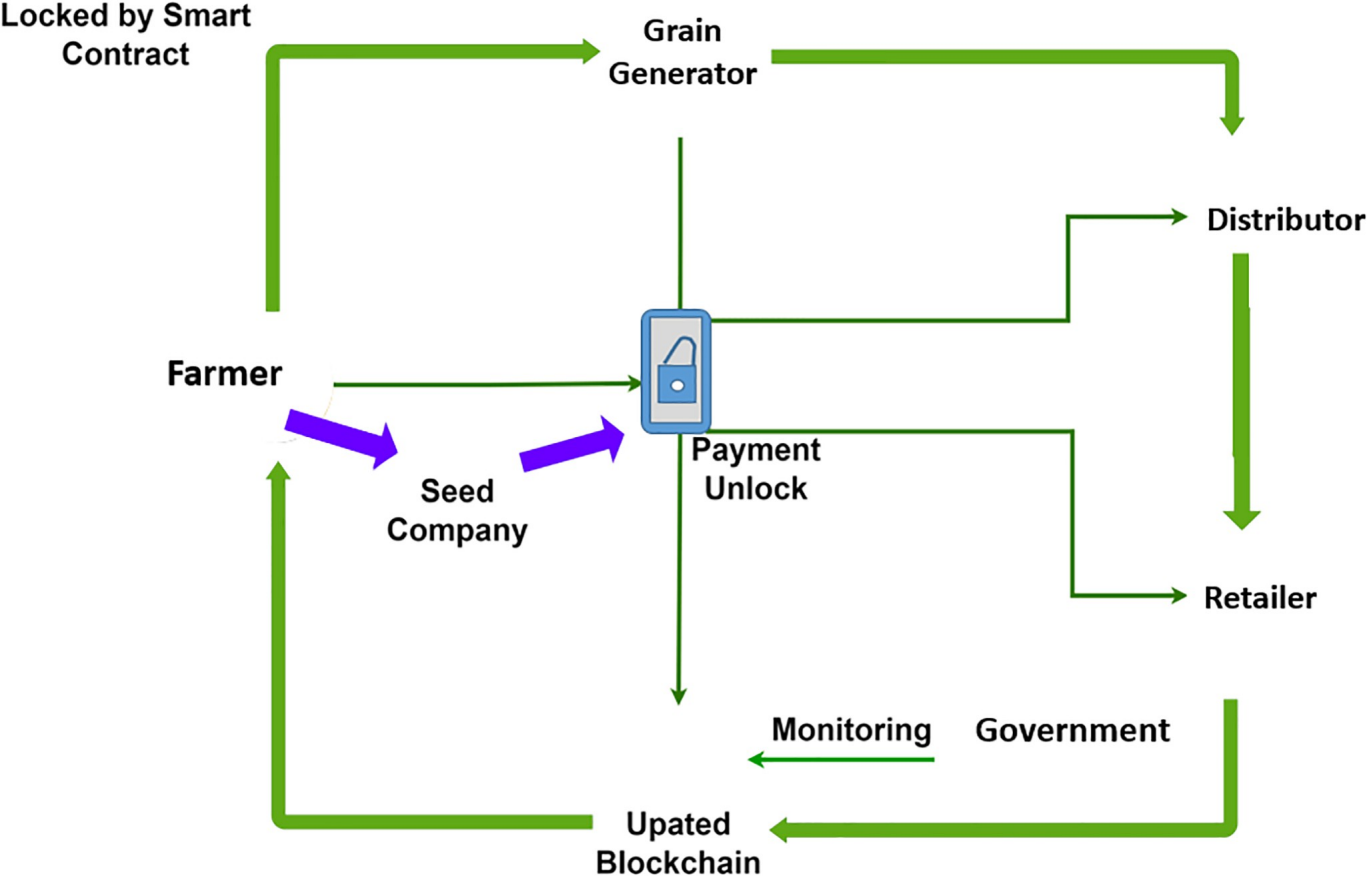
Supply chain of the proposed framework.

This study presents a framework for the wheat crop supply chain. The proposed framework utilizes state-of-the-art technology blockchain for providing a secure, transparent, and efficient traceability system of wheat crops from farm to consumer. Blockchain technology assures transparency in the supply chain by allowing all participants to trace the complete supply chain of the crop. The payment is secured by a smart contract whenever a farmer orders seeds from a seed supplier, which ensures that the transaction is secure. The payment data that are stored in the blockchain system cannot be tampered with or modified. In the whole supply chain, on-demand products in the supply chain move from one participant to another meanwhile payment transactions are secured and verified by smart contracts without centralized authority and a single point of failure. Fig 4 illustrates the components of the proposed framework.

### Architecture of proposed framework

**Fig 5:**
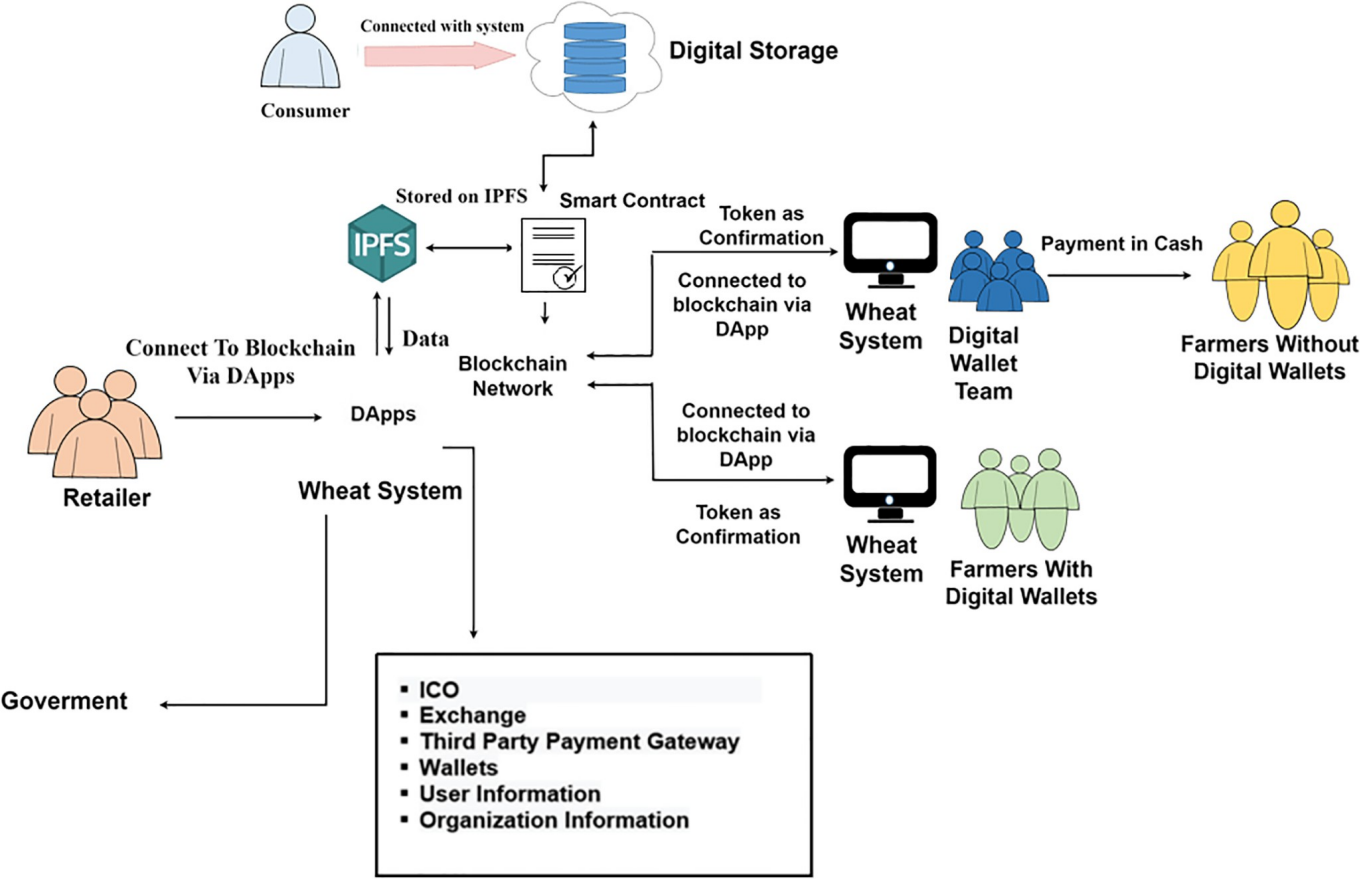
Architecture of the proposed system.

The high-level architecture of the proposed framework is presented in Fig 5. The main participants in the proposed framework are seed companies, farmers, grain generators, distributors, and retailers. One of the major components in the system is ‘dApps’ which consists of smart contracts, IPFS, distributed storage, a network of blockchain, and the web. Every participant in the wheat traceability system is connected with dApps to access the blockchain network. To join the system each participant must complete a registration form on the system and provide his/her personal information. Once the signup process has been completed, the blockchain system provides a public address that is unique for every user. Every user in the system is identified by this unique address.

For data transformation, IPFS is used between two components i.e., dApps and digital storage. Moreover, different users at different levels and management are also participants in the proposed system. The management system dApps consists of an initial coin offering, exchanges of initial coin offerings, and a gateway of third-party integration. The dApps also deal with the integration of different wallets because multiple wallets exist for various currencies. The proposed framework utilizes smart contracts to securely store records as well as statistical values of users. The IPFS technology is associated with digital storage and distributed networks. The consumer can purchase wheat crops through mobile dApp. All system transactions made by stakeholders are stored on the blockchain. When a customer receives a crop, farmers get a notification and one transaction is recorded on blockchain making it visible to other participants like distributors and retailers. The digital wallet team is assigned to assist farmers who have less knowledge about digital wallets and accounts.

Algorithm 1 presents a blockchain-based solution to enhance the traceability and transparency of the wheat supply chain. It introduces a new token called WC in order to record the transaction history between farmers, consumers, businesses, and all other stakeholders. Moreover, the smart contract automates the process of transactions, converts WC to fiat, and uses IPFS to store private data securely. Furthermore, the performance comparison of the framework with Ethereum and Bitcoin shows the efficiency of the proposed solution.

**Algorithm 1** Blockchain-based framework for wheat supply chain traceability.

*class WheatCoin*:

 *def_init_(self,owner,quantity)*:

  *self.owner=owner*

  *self.quantity=quantity*

*class SmartContract*:

 *def_init_(self)*:

  *self.transactions = [ ]*

 *def add_transaction(self,sender,receiver,amount)*:

  *self.transactions.append({‘sender’:sender,‘receiver’:receiver,‘amount’:amount})*

*class Blockchain*:

 *def_init_(self)*:

  *self.chain = [ ]*

  *self.current_transactions = [ ]*

 *def create_block(self,proof)*:

  **# Create a new block in the blockchain**

  *block={*

   *‘index’:len(self.chain)+1*,

   *‘timestamp’:time()*,

   *‘transactions’:self.current_transactions*,

   *‘proof’:proof*

   }

  *self.current_transactions = [ ]*

  *self.chain.append(block)*

  *return block*

 *def add_transaction(self,sender,receiver,amount)*:

  *self.current_transactions.append({‘sender’:sender,‘receiver’:receiver,‘amount’:amount})*

**# Initialize blockchain and smart contract**

*blockchain = Blockchain()*

*smart_contract = SmartContract()*

**# Simulate transactions**

*farmer_wallet=‘farmer_address’*

*business_wallet=‘business_address’*

*consumer_wallet=‘consumer_address’*

**# Farmer registers batch of wheat**

*blockchain.add_transaction(None,farmer_wallet,100)*  *# Mint 100 WC for farmer*

**# Business buys wheat from farmer**

*blockchain.add_transaction(farmer_wallet,business_wallet,50)*

**# Consumer buys wheat from business**

*blockchain.add_transaction(business_wallet,consumer_wallet,25)*

**# Create a new block on the blockchain**

*blockchain.create_block(proof=12345*

**# Display blockchain in transactions and details**

**for** *block in blockchain.chain* **do**

 *print(“Block:”,block[‘index’])*

 **for** *tx in block[‘transactions’]:* **do**

  *print(“Transactions:”,tx)*

 **end for**

**end for**

### Economic model

**Fig 6:**
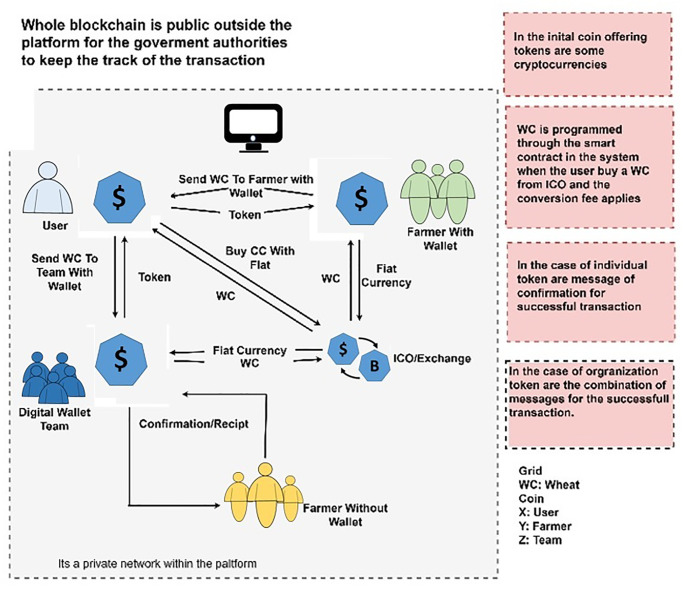
Economic model.

This study also provides an economic model for a safe wheat traceability system that is entirely traceable, transparent, and reliable for both authorities and participants. The proposed economic model is shown in Fig 6. A new digital token WC is introduced for the protection of the system from illicit actions. Table 2 illustrates the fundamental properties of the proposed WC.

**Table 2:** Parameters and values of the wheat coin.

Parameter	Value
Symbol	Wheat Coin
Coin Supply	100M
Network Type	ERC20.
Price	1 WC = 100 USD
Currencies	PKR,Dollar, Euro

### Security analysis

For the security testing of the proposed system, we tested the smart contract by applying different famous smart contract attacks such as exploit, access control, and reentrancy attacks. In the past, different hackers used these attacks to destroy well-known smart contract-based systems. So, we applied these attacks one by one for the security analysis of the system and the proposed system performed well indicating the security of the proposed system.

The total supply of wheat coins is set to 100 million, and each coin is equivalent to 100 United States dollars (USD). It is not mandatory to buy a whole coin, users can purchase coins in decimals according to their needs. For instance, if someone needs $10 of wheat then he should have to purchase 0.1 WC. The benefit of using such cryptocurrency is that the amount of transaction does not rely on financial values, and in this scenario payment of $10 is added to the system against the private key of the participant, and this transaction is part of blockchain in terms of UTXOs. By using the ERC20 protocol of ICO generation of the wheat coin is developed using the smart contract. Any user can purchase wheat coin using any currency that is either fiat currencies like USD, Pakistani Rupees (PKR), Euro (EUR), or some other cryptocurrency such as Bitcoin, Binance, or Ethereum. As a result, the participants get tokens in terms of wheat coins that are added to their wallets. There is no exchange fee or tax while purchasing WC due to the decentralized nature of blockchain.

The purchasing of the wheat crop can be made in two different scenarios. There are two methods defined for participants to trade in WC. The first option is that the user can purchase WC first, then WC can be used for crop purchase, and payment from the farmer account is transferred to the farmer wallet and this transaction has been done through the wallet address. The second option is that farmers can send payment to the digital wallet team, and then the team can pay a cash amount to farmers who do not have a digital wallet. In a nutshell, the proposed system is hybrid. For the participants, the network of blockchain is private but for officials like the government authorities, blockchain is public. So that the government can track every transaction to control prices of the crop from dishonest people to provide a quality product to the consumer at appropriate rates.

The wheat coins which are released to users are backed with the currency. The amount the user pays in the dollar will get the equivalent wheat coin according to their amount. Moreover, the price of the wheat coin will be fixed which is $100 as mentioned in Table 2. It will not be changed.

### Token division

The startup generation distribution of wheat coin is as follows: initial tokens of sixty percent (60%) are assigned for ICO token sale, and thirty percent (30%) are assigned to authorities for the development of the wheat coin. Moreover, for the enhancement of the ecosystem 10% token is assigned. The total supply of WC has been fixed and no new coins can be generated.

### Fee for conversion

In the proposed framework following fees have been defined in terms of the long-term sustainability of wheat coins. For each of the transactions, a specific transaction fee amount is to be charged, which is used for the miner’s incentives. Based on expenses, a fixed percentage of the fee is charged while the transaction is made through an exchange like KUcoin, or Binance and this fee varies from exchange to exchange depending on digital or cryptocurrency. Moreover, a fixed percentage of the amount is charged as a fee for system maintenance. The WC’s self-sustainability is ensured by the cash flow generated by these administrative expenses.

### Workflow of proposed framework

**Fig 7:**
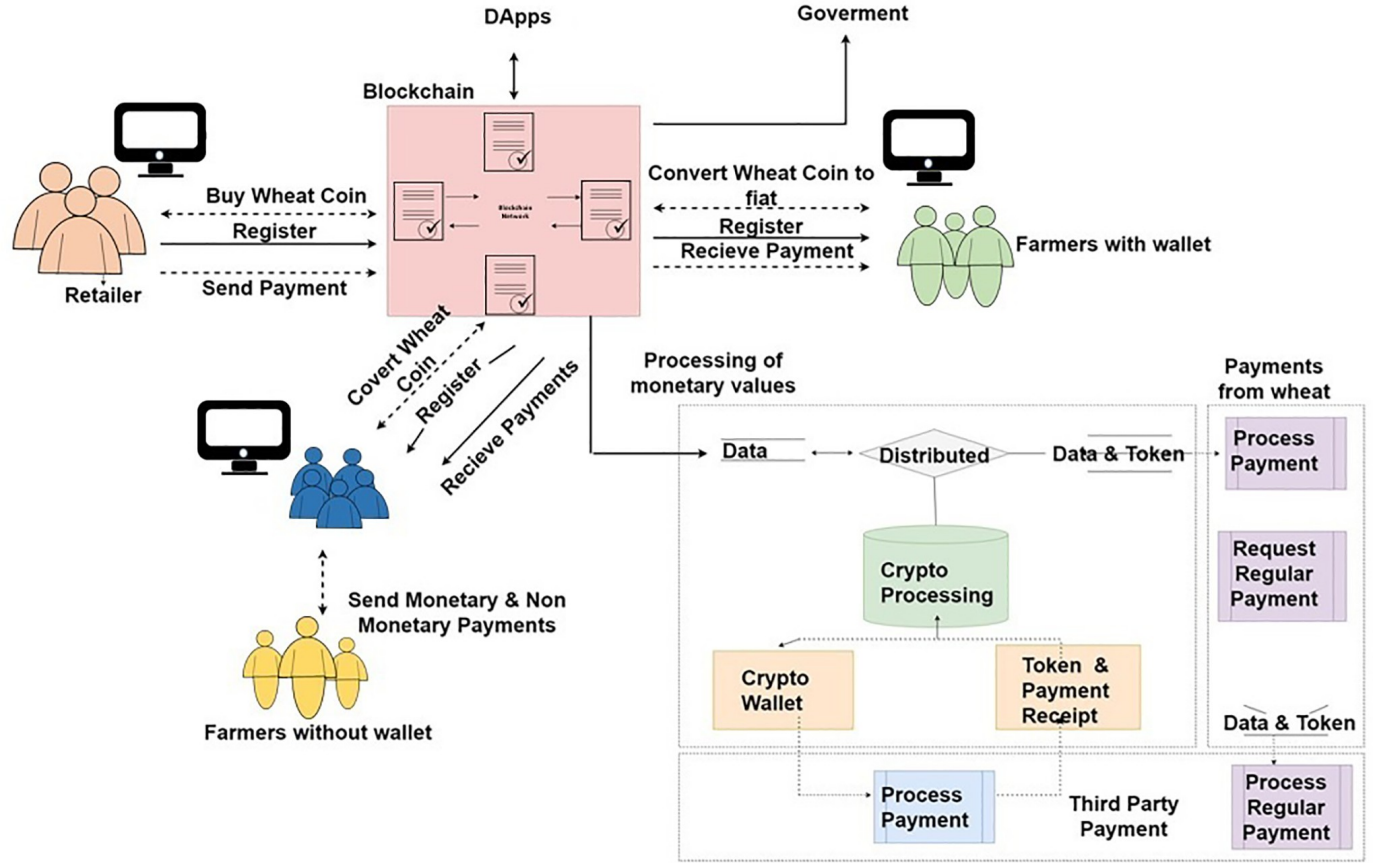
Flow of the proposed framework.

The workflow of the proposed framework is illustrated in Fig 7. Through the wheat management system, the application users are connected with dApp. Every user is connected to the system and has their own public address where they can handle their profiles as well as manage their wallets. ICO and exchanges are running at the backend of the platform. By using the front-end application of the system, participants are connected with exchange and ICO. When one transaction is completed the remaining UTXOS has been available in the wallet of the retailer and the user can transfer these UTXOs to farmers or other participants easily. Farmers and other participants are directly connected through blockchain using exchange and ICO and can exchange their currency. In case of transfer through the team, the team exchanges fiat money to help farmers and retailers make online transactions. For the authentication of users and management of smart contracts the administration handles and generates sheets, as well as controlled policies of token distribution, and these policies explain tokens in ICO. ICO of the wheat coin is handled through smart contracts that include equities of the wheat coin. Moreover, the rate of the wheat coin against different currencies in exchange is defined based on the smart contract.

### Proposed framework layered structure

**Fig 8:**
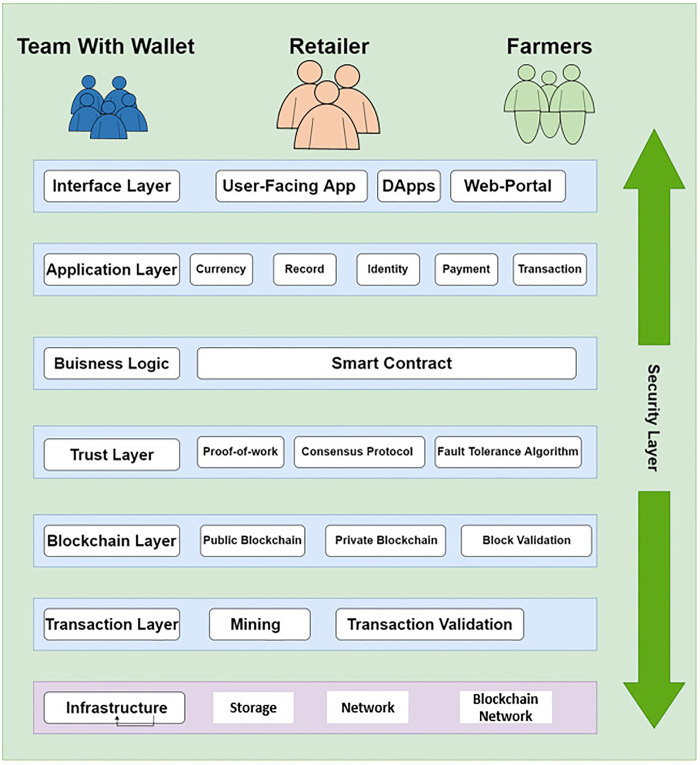
Layered architecture of the proposed framework.

The proposed framework is a layered-based architecture style system, layer structure is illustrated in Fig 8. The system has been divided into eight layers named as an interface layer, application layer, business logic layer, trust layer, blockchain layer, transaction layer, infrastructure layer, and security layer. The working of each layer is as follows.

#### Application layer

In the system, the application layer is responsible for keeping online records, verifications of identities, and the data of transactions. Moreover, the form of the smart contract application layer connects the business logic layer with the interface layer.

#### Business logic layer

The business logic layer is responsible for the terms and regulations of the system because this layer has smart contracts. That is why this layer is considered a smart contract active database that includes all communication, contract, and execution rules.

#### Trust layer

The trust layer contains the smart contract’s security analysis, formal verifications, and consensus methods like proof-of-work and Byzantine fault tolerance. For newly added block verification, as well as consensus protocol of transaction trust layer is responsible.

#### Blockchain layer

The blockchain layer holds the basic information of the distributed ledger, as well as the hashes of transactions conducted by participants against their public and private key addresses.

#### Transaction layer

The transaction layer is responsible for transactions that are initiated by a smart contract or a donation management system participant.

#### Infrastructure layer

The infrastructure layer is made up of a peer-to-peer network for the distribution, and verification, and aims to transfer transactions of the Ethereum blockchain. When a transaction is completed, it is broadcast to all nodes in the network, and each node verifies the transaction using predetermined parameters. The validated transaction is then placed in the blockchain.

#### Security layer

The security layer is a very important layer in the blockchain network. Blockchain is susceptible to a variety of security threats, the most common of which is the 51 percent attack. This layer consists of many security protocols, as well as connected and works with the system. The security layer also consists of the administrative roles that help to maintain system integrity. The participants including the farmers, distributors, and retailers have wallets of crypto. To interact with these wallets they use an interface that consists of DApp or web portals.

#### Trading system

Trading in the system is done through decentralized exchanges such as Pancake Swap and Uniswap. The smart contracts of these exchanges are tested, trusted, and audited by audit companies. These exchanges will be used to convert the fiat into the wheat coin.

## Experiments and results

This section contains a simulation of real-time use cases of the implemented framework and results of the wheat crop supply chain framework.

### Experiment setup

For deployment and performance evaluation of the proposed framework, real-time scenarios are used. In the proposed framework data transfer and transaction are done between entities by using a test network smart chain and through Meta Mask. To set up HTTP requests, Postman is used to connect with the blockchain. Two functions ‘Validate_block’ and ‘Access_chain’ are used for the performance evaluation of blockchain. By using the web3 python library, 65000 transactions are performed. These transactions have been used as data sets for the evaluation of the proposed system. Performed experiments for evaluation are as follows:

1. Calculate the response time of the longest chain retrieving data in milliseconds,
2. Latency of adding new blocks and data comparison with other well-known blockchains,
3. Latency and block confirmation comparisons for transactions, blocks, and data,
4. Gas fees for transactions are compared to other proposed frameworks,
5. For performance evaluation, a comparison with other blockchains is made.

### Response time to retrieve blocks

**Fig 9:**
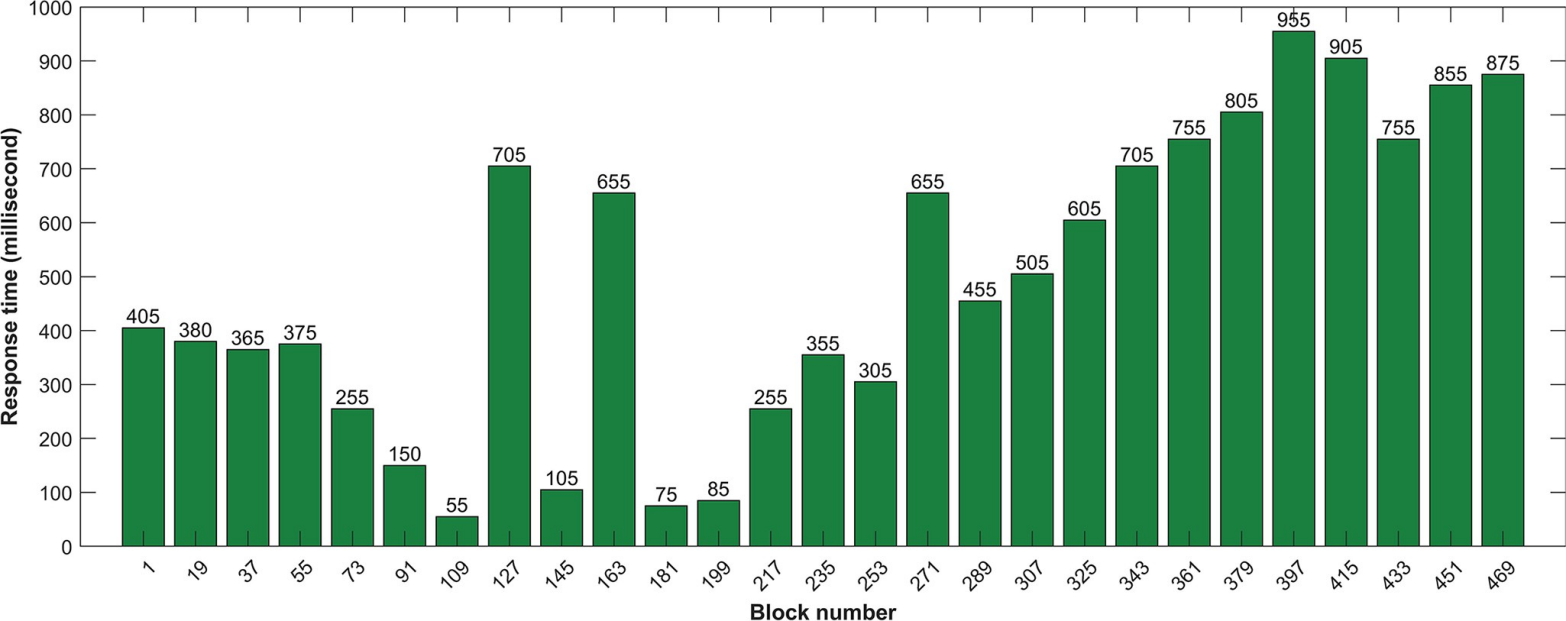
Response time to retrieve the longest chain.

For retrieving data on different blocks on the blockchain ‘AccessChain’ function is used. By using this function we retrieved some specific types of data from different blocks and calculated the time for accessing data by using the time stamp. At the initial stages, the time latency of retrieving block data is almost 400 milliseconds then it drops down to 91 milliseconds and after some time it increases to 950 milliseconds. The change in the response time to access data happens due to the decentralized nature of node division. Retrieving data from different nodes leads to different time delays in the system. When the block size increases, there is a significant increase in retrieving time, as shown in Fig 9.

### Time to add new blocks

**Fig 10:**
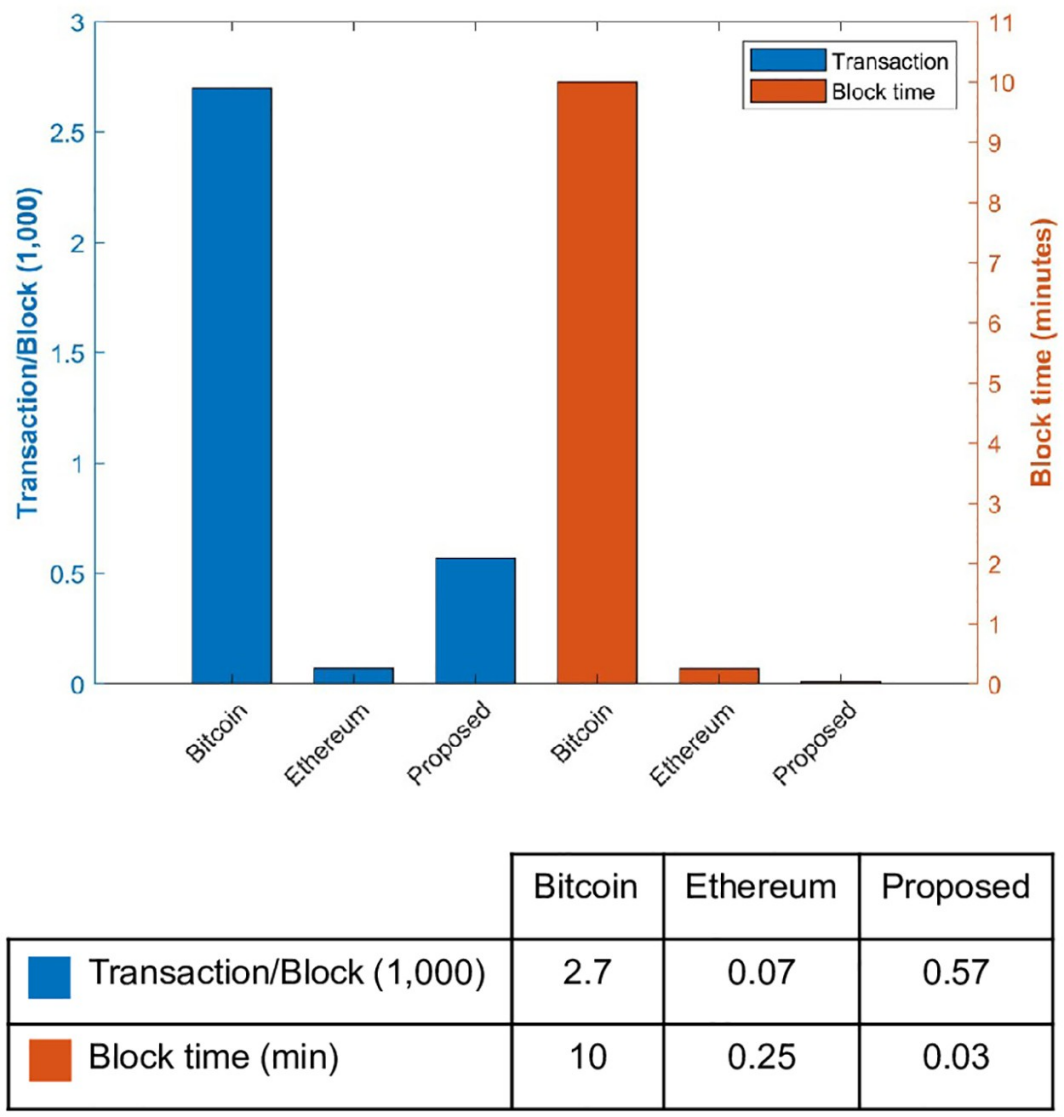
Block time for different approaches.

Experiments are performed to analyze the performance regarding the time needed to add new blocks. When we compare the proposed system with other available solutions, the proposed solution took very little time to add a new block. Blockchain of Bitcoin takes 10 minutes while Ethereum takes approximately 15 to 20 seconds to add a new block. The proposed system, on the other hand, takes only 3 seconds to add a new block. The performance of latency to add new blocks to the blockchain is shown in Fig 10. These fast transactions make our proposed framework more efficient and it can verify new coming transactions faster. The proposed solution adds 0.57k transactions every 3 seconds which is very fast as compared to both Bitcoin and Ethereum blockchain which adds 2.7k and 0.07k transactions per block as their time to add new block is slow.

### Time for verification and transactions per block

**Fig 11:**
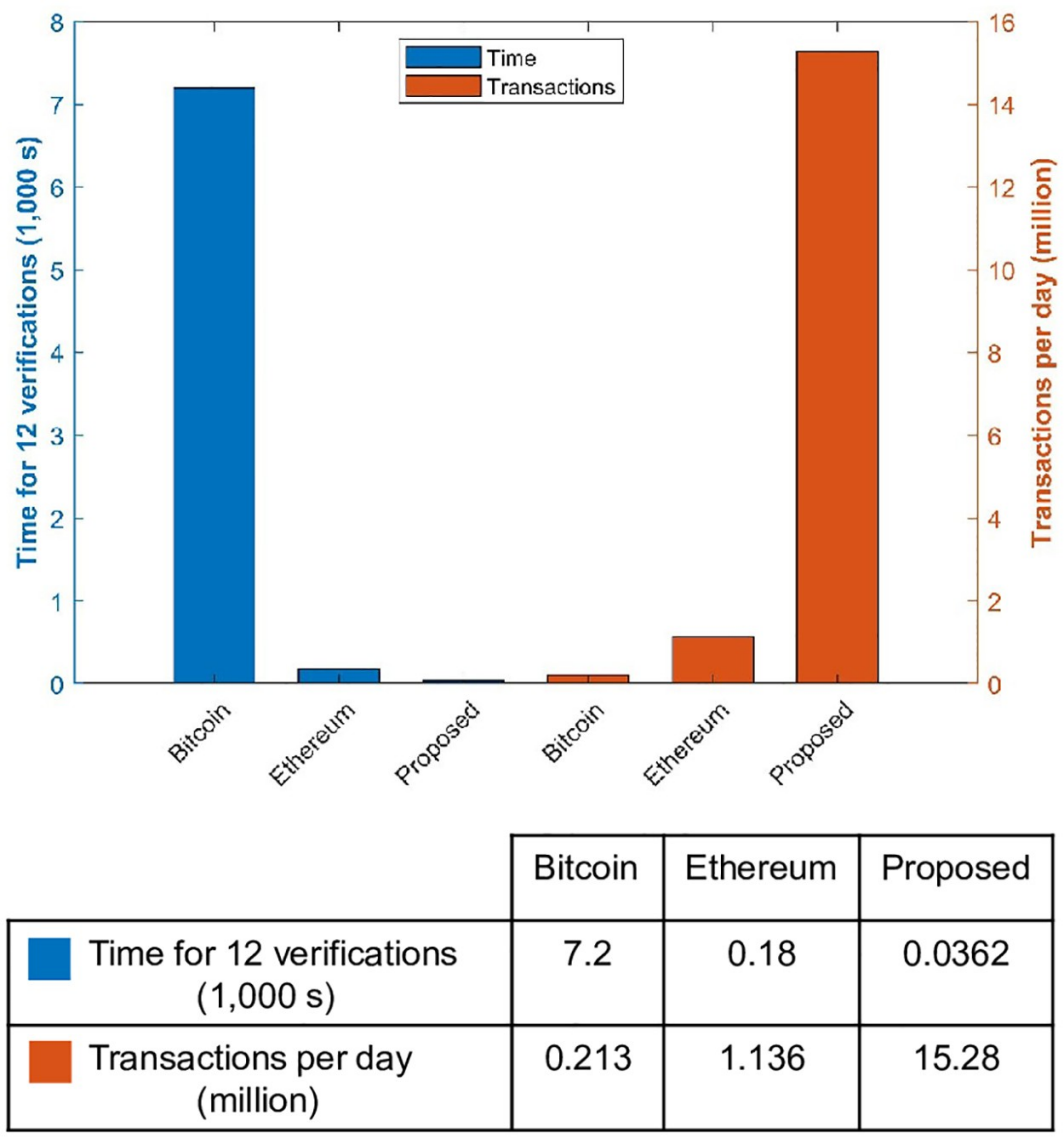
Verification time and transactions per day for different approaches.

Another important parameter that is used to investigate the performance of the proposed system in comparison to Bitcoin and Ethereum is the time needed to verify a transaction. Also, the number of transactions per block is used for the same purpose. The performance of the proposed system, Bitcoin and Ethereum is shown in Fig 11.

Verification time is shown for 12 transactions as it is more appropriate to show the values due to small values for the proposed system. The time for 12 verification using the Bitcoin blockchain is 7.2 kilo seconds and for the Ethereum blockchain, it is 0.18 kilo seconds while the proposed framework carries out the verification of 12 transactions in merely 0.0362 kilo seconds which is substantially lower than the Bitcoin and significantly less than the Ethereum. Regarding the number of transactions per day, Bitcoin performs 0.213 million transactions per day and Ethereum performs 1.136 million transactions per day. Better than both the existing systems, the proposed system performs 15 million transactions per day.

### Performance for gas fee

**Fig 12:**
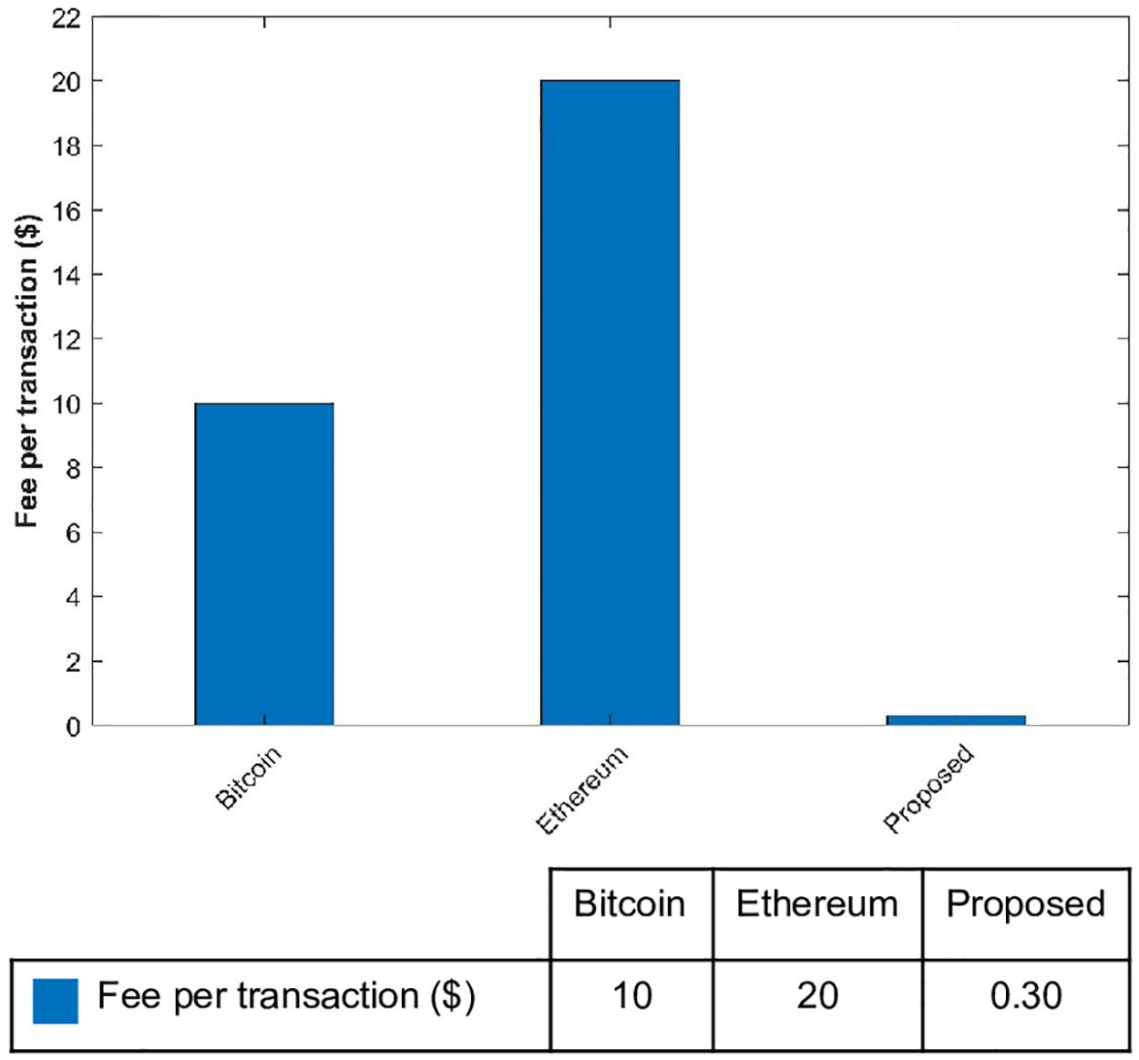
Average gas fee per transaction for different approaches.

Lastly, we evaluate the performance of the proposed framework in terms of gas fees with existing blockchain systems. A comparison of performance regarding gas fee is given in Fig 12. Bitcoin charges $11 for each transaction as a gas fee while Ethereum uses $30 for each transaction. If the Ethereum system is busy or has a potentially higher number of transactions in the pool, it charges a high fee. In comparison to both Bitcoin and Ethereum, the proposed model charges a lower fee of only $0.10 for a transaction which makes it more efficient compared to other systems.

### Security analysis

For the security testing of the proposed system, we tested the smart contract by applying different famous smart contract attacks such as exploit, access control, and reentrancy attacks. In the past, different hackers used these attacks to destroy well-known smart contract-based systems. So, we applied these attacks one by one for the security analysis of the system and the system performed well indicating the security of the proposed system.

### Comparison with existing approaches

In the end, a performance appraisal of the proposed system is made with existing systems to show its superior performance and supremacy over state-of-the-art approaches. For this purpose, [12, 18, 25] are selected. [18] uses blockchain technology to propose a supply chain management system and is suitable for comparison. Similarly, the Ethereum blockchain-based system is presented in [12] for traceability involving the use of smart contracts. Results given in Table 3 indicate that the proposed system shows significantly better performance as compared to existing systems regarding the time needed for adding the new block, the capability of performing transactions per block, the time needed for transaction verification, number of transactions per day by the system and the fee charged for each transaction.

**Table 3:** Comparison with existing blockchain-based approaches.

Ref.	Block time (min)	Transactions per block (1,000)	12 verifications time (1000 s)	Per day transactions	Transaction fee ($)
[25]	11	2.8	7.3	0.215	$10.2
[18]	0.267	0.08	0.19	1.138	$20.5
[12]	0.071	0.251	0.182	1.14	$30
Proposed	0.035	0.58	0.037	16.01	$0.35

### Discussion

Wheat crop supply chain management has significant importance for human survival and should be efficient in providing different features like traceability, security, transparency, etc. With recent events of war between Russia and Ukraine, the shortage of wheat crops and scarcity of appropriate supply chain has been highlighted. To resolve the above-mentioned issues, blockchain-based supply chain management systems can be designed. This study presents a similar endeavor to overcome these challenges and presents a secure, decentralized, and efficient wheat crop supply chain management system. The presented idea in this paper is simple yet important. Experimental results show its potential, however, a lot of work is still required in terms of security and efficiency. Technically, the proposed system helps farmers and the government to make wheat crop sale and purchase secure by using blockchain technology. Blockchain technology removes the centralization from the system and makes the overall system transparent. However, the security and overall system design still need to be improved further.

## Conclusion

The current system of the wheat crop supply chain does not possess traceability, reliability, and security capabilities. In addition, it does not provide transparency which results in high-cost selling of crops to consumers, often caused by the artificial shortage of stock. An efficient, secure, and transparent supply chain management system is needed to resolve these issues. This study proposes a blockchain-based framework for the wheat crop supply chain that ensures traceability, transparency, and security. The wheat coin is introduced to keep track of transactions, in addition to the initial coin offering, crypto wallets, and economic model for the proposed system. The proposed framework is built on the Binance smart chain network and provides faster transaction verification with fewer transaction fees than current blockchains. The smart contract and digital wallets are used to design the system. The IPFS system is used to store encrypted information of stakeholders. Experimental results indicate that it performs significantly better than existing systems in terms of latency for adding new blocks, transactions per minute, average gas fee for transactions, and transaction verification time. This research provides a framework for supply chain management with a focus on the most common problems of the supply chain of wheat. Moreover, after the implementation of this proposal, the income of the farmer will be increased. Besides this, if the Government wants to decrease the fee of the system, they have to deploy their own nodes. However, it may lead to centralization and control of the system by their choice. The developed system is designed for financial transactions and can become the target of hackers, however, blockchain provides an enhanced level of security. In the future, we intend to investigate more specific issues related to the supply chain, as well as, develop a mobile application to increase system reachability and use. Also, we intend to work on the security of the system as this system is designed for financial transactions it is very important to secure the system from different hacking attacks.
